# Quality of life and level of post-traumatic stress disorder among trauma patients: A comparative study between a regional and a university hospital

**DOI:** 10.1186/s13049-018-0507-0

**Published:** 2018-06-01

**Authors:** F. B. Danielsson, M. Schultz Larsen, B. Nørgaard, J. M. Lauritsen

**Affiliations:** 1 Department of Orthopaedic Surgery and Traumatology, Odense University Hospital, Odense, Denmark; 2 Department of Orthopaedics, Kolding Hospital, part of Lillebaelt Hospital. Odense Universitetshospital Sdr, Boulevard 29, DK5000 Odense C, Denmark; 3 Department of Clinical Research, University of Southern Denmark, Odense, Denmark; 4 Department of Public Health, University of Southern Denmark, Odense, Denmark

**Keywords:** Quality of life, Severe injuries, Trauma outcome, PTSD, EQ-5D

## Abstract

**Background:**

The aim of this study was to assess outcome in long-term quality of life (QoL) and post-traumatic stress disorder (PTSD) among adult survivors of trauma. Secondary aim was to compare levels of the outcome with injury severity and specialization level of two trauma centres.

**Methods:**

A retrospective study included patients received by the trauma response teams at two hospitals in 2013 aged 18 or more at follow-up. We assessed QoL and PTSD with one mailed questionnaire to each patient at either 12 or 24 months of follow-up. Health status was measured by EuroQol EQ-5D and the Glasgow Outcome Scale. PTSD symptoms were classified according to the Post-Traumatic Stress Disorder Checklist (PCL) and Diagnostic and Statistical Manual of Mental Disorders, 4th Edition (DSM-IV).

**Results:**

A questionnaire was mailed to 774 patients at end of 2014 or early 2015, 455 were included for analysis; median age 44 (IQR 25–57; 68% male); median NISS 9 (IQR 2–17); At follow-up 24% (95% CI 20–28) reported a EQ index score value equivalent to the lowest 2.3% in the Danish population norm. Probable PTSD was present in 19% (95% CI 13–27) of patients with severe injuries (NISS> 15), and 23% (95% CI 19–28) of those with NISS < 15.

**Conclusion:**

Severe trauma has substantial impact on QoL and PTSD assessed at 12–24 months after the trauma. The QoL was well below the Danish population norm. The presence of PTSD was independent of injury severity. Trauma Centres should consider to include this as part of the treatment principles.

## Background

Injury is the leading cause of death in people aged 5–44 in high-income countries, and the leading cause of death and disability for all age groups below 60 worldwide [1]. This amounts to 10% of global deaths [2]. Furthermore, injury represents a major cost to families, the health care system and society [3].

Major improvements in the quality of trauma care have been made in recent decades, and this has reduced the number of potentially avoidable deaths [4]. Injury severity is most often classified using the Abbreviated Injury Scale (AIS) as a basis for the Injury Severity Score (ISS) and the New Injury Severity Score (NISS). These scores are used in the assessment of overall injury severity in patients with multiple injuries. Injury severity classification is considered to be a fundamental component of trauma outcome research and quality assessments, and thus a crucial variable in modern trauma registries. Improvements in trauma care, combined with improvements in injury prevention and prehospital care, have increased the probability of surviving a major trauma [5]. Because survival after major trauma is improving, attention can now be directed towards quality of life after survival [6]. Few studies have been conducted in Scandinavia on long-term QoL outcomes [7–9].

In the assessment of long-term trauma outcomes for survivors, not only physical impairments but also mental health needs to be considered. One important mental outcome entity, as demonstrated in many trauma outcome studies, is post-traumatic stress disorder (PTSD) [9–11].

It is well-known that trauma patients suffer from impaired QoL after major trauma [8, 12–16]. However, available studies have focused on the most severely injured patients (ISS > 15) [9, 15, 17, 18].

Many trauma survivors are young, and their daily activities can be greatly, and sometimes permanently, impacted by the consequences of trauma. Therefore trauma registries should also include data on injury-related disability, as recommended more than a decade ago [19]. The Southern Denmark trauma database contain information from the Utstein template for major trauma [20], but this does not include QoL measures.

Studies of long-term QoL in trauma survivors have identified the Injury Severity Score (ISS) [18, 21], extremity injury [12, 18], age [21, 22], female gender [13], lower socioeconomic status, and living alone as independent predictors of poor quality of life after severe trauma [18]. Furthermore, severely injured patients are known to face a major risk of PTSD [17], which has not been assessed in a Scandinavian trauma setting. PTSD has been reported to predict low QoL and be most commonly present in severely injured patients [23]. We found no study including all patients received by a trauma team, regardless of Injury Severity Score.

Therefore, the primary aim of this study was to assess the long-term quality of life and symptoms of post-traumatic stress disorder (PTSD) of survivors of trauma and quality of life (QoL) in relation to injury severity and as a secondary aim to study variation between a university and a connected regional centre.

## Methods

### Setting

The study was conducted in Denmark for a region of 1.2 million in two centres. The university trauma centre serves as the primary facility for a population of around 500,000, and as a secondary referral centre for the whole region. One regional trauma centre serves as the primary facility for a well-defined geographical area with a population of around 300,000. All transfers from the regional centre go to the university centre in this study, when there is an immediate need for higher level treatment.. Two other primary centres were not included.

The study was approved by the Danish Data Protection Agency (Journal no. 13/32033). Data protection met the standards set by Danish law, which allows patients to be contacted for follow up. The study complied with the ethical and legal regulations in Denmark for clinical studies of this nature.

### Study population and data collection:

We included a retrospective cohort consisting of all consecutive patients received by the trauma response teams at the two trauma centres for the full calendar year 2013. Systematic review of patient records and ongoing quality-assureance (completeness and content) for inclusion in the Southern Denmark trauma database secures a consecutive patient series of all patients received by the trauma teams, which is an indication that there was a potential threat to life at the scene. Trauma teams were activated based on a structured criteria for the potential impact of the incident. Data on mortality and current addresses were obtained from the Danish Civil Registration System [24].

**Fig. 1:**
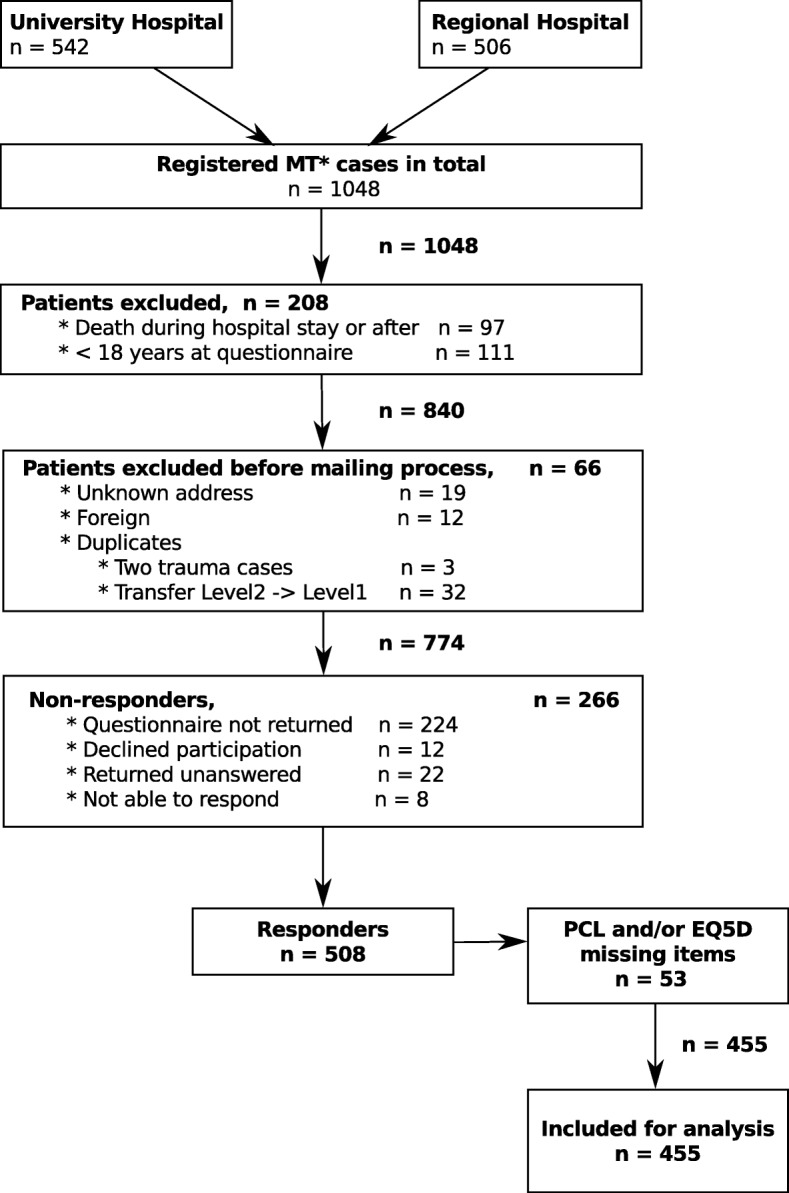
Flow chart of eligible patients. *Multi Trauma (MT): defined as received by trauma response team. PCL: Post-Traumatic Stress Disorder Checklist. EQ-5D: Euro QoL 5D questionnaire

To comply with regulations only patients aged 18 or older, alive at follow-up and with a Glasgow Outcome Scale (GOS) ≥ 3 were contacted via the questionnaire [25]. Detailed numbers are shown in fig. 1. Questionnaires were sent from November 2014–January 2015 such that patients were contacted either at 12–17 months or 18–24 months after the injury. In addition to QoL and PTSD questionnaires, questions on other aspects were formulated in accordance with The Danish National Health Survey from 2013 [26].

### Quality of life

Quality of life was assessed using the European Quality of life questionnaire (EQ-5D) [27], including a Visual Analog Scale (VAS; 0–100 scale) and the five-question items in version EQ-5D-5 L. The questionnaire consists of the following five dimensions: mobility, self-care, usual activities, pain/discomfort, and anxiety/depression [27, 28].

Each dimension has five levels: no problems, slight problems, moderate problems, severe problems, and extreme problems. The ratings for the five dimensions were converted into a single index (the utility score) using population-based preference weights from the Danish population norm [29].

### Assessment of PTSD

The civilian version of the Post-Traumatic Stress Disorder Checklist (PCL) was used [30, 31]. The PCL is a 17-item checklist, in which each item has five levels and was assumed positive when scored at level 3 or higher [32]. To diagnose PTSD according to DSM-IV, patients must fulfil 4 criteria (A + B + C + D). Criterion A refers to the occurrence of a stressful event involving life danger and intense fear, horror, and helplessness. Symptoms are described by groups B (requiring ≥1 intrusion symptoms), C (requiring ≥3 avoidance/numbing symptoms), and D (requiring ≥2 hyperarousal symptoms). For this study, we defined PTSD positive as DSM-IV (A + B + C + D) [32] in combination with an overall minimum PCL sum of 37 [33]. It has been translated into Danish and has been used in several studies [34, 35]. Validation studies have demonstrated good psychometric properties, both internationally and in a Danish setting [33].

### Statistical analysis

Demographic and clinical data are presented as either means + 95% confidence interval (CI) or medians + interquartile range (IQR), if not normally distributed for continuous variables. Categorical variables are presented as either proportions with CIs or percentages. Statistical tests were performed according to type of variables and comparisons (Chi-square, *t*-test, Mann–Whitney *U*-test, two sample equity of proportions).

The Danish population norm data was used to compare the health status of the patients included in this study. The EQ-5D index score were compared between groups. When QoL was examined, the scores were dichotomized. Low QoL (Low EQ) was defined as being lower than 2 standard deviations below the Danish national norm, and thereby includes the 2.3% with lowest QoL in the Danish population. The proportion of the study population with a QoL equal to the low EuroQoL score in the Danish population was then calculated.

Data entry with double entry verification was performed with EpiData software (www.EpiData.dk) and analysis using STATA (www.stata.com).

## Results

### Response rates

In 2013, 1048 patients were received by the trauma team at either the university hospital or the regional hospital, and were thus entered into the trauma registry. Approximately, the same number of patients were treated at each hospital (542; 506). Of the 542 patients received at the university hospital, 32 were acute cases transferred from the regional hospital. Details of the inclusion is shown in fig. 1.

A total of 508 (66%) returned the questionnaires and agreed to participate. Differences between responders and nonresponders are presented in Table 1. Nonresponders (*n* = 266) were younger than responders and more often males (340 vs 202, *p* = 0.009). More responders than nonresponders had a NISS higher than 15 (346 vs 211, *p* = 0.001).

**Table 1:** Characteristics of trauma patients, in relation to response and composition of those included in analysis

Variables		Comparison			
		Included for analysis	Responders (returned questionnaire)	Nonresponders	*p*-value
Total		455	508 (65.6%)	266 (34.4%)	
Hospital trauma level					*p* = 0.108*
University trauma centre		235	262 (68.4%)	121 (31.6%)	
Regional trauma centre		220	246 (62.9%)	145 (37.1%)	
Study groups					*p* = 0.000*
12–17 months		280	312 (71.2%)	126 (28.8%)	
18–24 months		175	196 (58.3%)	140 (41.7%)	
Sex					*p* = 0.009*
Male		309	340 (62.7%)	202 (37.3%)	
Female		146	168 (72.4%)	64 (27.6%)	
Age at trauma - Grouped					*p* = 0.000*
16–39 years		192	205 (54.1%)	174 (45.9%)	
40–59 years		166	182 (73.4%)	66 (26.6%)	
60+ years		97	121 (82.3%)	26 (17.7%)	
Other characteristics					
NISS**					*p* = 0.001*
NISS < 15		316	346 (62.1%)	211 (37.9%)	
NISS > 15		139	162 (74.7%)	55 (25.3%)	
Traffic, % (n)**		258	290 (62.8%)	172 (37.2%)	*p* = 0.041*
ICU**		71	81 (72.3%)	31 (27.7%)	*p* = 0.107*
Glasgow Outcome Scale**					*p* = 0.003*
GOS 3		37	46 (71.9%)	18 (28.1%)	
GOS 4		97	114 (76.0%)	36 (24.0%)	
GOS 5		280	302 (61.5%)	189 (38.5%)	

Characteristics in relation to response and composition of those included in analysis

All patients *n* = 774 – included in analysis were those with completed EQ5D AND PCL: *n* = 455*chi^2^ within each variable except traffic and ICU, where *p*-values was compared to non traffic /ICU

**Groups not equal to total (*n* = 774) due to missing data

*NISS* New Injury Severity Score, *ICU* Intensive care unit stay > 2 days

*GOS 3* severe disability, *GOS 4* moderate disability, *GOS 5* good recovery

The proportion followed up at 12–17 months at the two hospitals was the same (university hospital (0.63; CI 0.57–0.69), regional hospital (0.63; CI 0.56–0.68)). The proportion followed up at 18–24 months at the two hospitals was the same (university hospital (0.37; CI 0.31–0.43), regional hospital (0.37; CI 0.32–0.44)). The response rate in the 12–17-month follow-up group was higher than in the group contacted after 18–24-months.

All items in EQ-5D were answered in 495 questionnaires (97%) and in 461 PCL questionnaires (91%) For 455 patients a completed EQ-5D and PCL questionnaire was received. These were included for analysis.

### Pattern of injuries

The most common mechanism of injury was traffic injury. Further analyses of more specific injury types were not conducted due to lack of detailed data.. The median NISS for all respondents was 9 (IQR 2–17), and more than 30% of recruited patients had major trauma (NISS > 15). As expected, median NISS scores at the university hospital were higher than at the regional hospital (12 vs 5, *p* = 0.000) (Table 2).

**Table 2:** Transferred trauma patients from regional hospital to university hospital that survived and completed questoinnaires

Transferred trauma patients from regional hospital to university hospital that survived and completed questoinnaires				
	All included for analysis *n* = 455	Transferred *n* = 12	University Hospital excluding transferred *n* = 223	Regional Hospital excluding transferred *n* = 220
ISS, median (IQR)	6 (2–13)	19 (12–26)	9 (4–17)	5 (1–9)
NISS, median (IQR)	9 (2–17)	22 (13–30)	12 (4–22)	5 (2–12)
MAIS, median (IQR)	2 (1–3)	3 (3–5)	3 (2–3)	2 (1–3)

Included for questionnaires at the university hospital but were initially received at the regional hospital

*ISS* Injury Severity Score, *NISS* New Injury Severity Score, *MAIS* Max AIS score

In multivariate analysis, injury localization (spinal cord injury and lower extremity injury) was negatively associated with EQ-VAS, EQ index, and PTSD symptoms. The proportion of trauma patients with internal head injuries and an AIS score > 1 was 0.27 (CI: 0.23–0.31). The proportion of spine injuries with an AIS > 1 was 0.20 (CI: 0.17–24) and the proportion of lower extremities injuries with an AIS > 1 was 0.15 (CI: 0.12–0.19).

### Health status measurement

The trauma population reported poorer self-rated health than the population norm: “Good, very good or excellent health” 0.654 (CI: 0.608–0.698) (norm: 0.852 (CI: 0.850–0.853)) [26]. Pain and discomfort: “High levels of pain and discomfort” 0.586 (CI: 0.539–0.633) (norm: 0.376 (CI: 0.374–0.378)). This tendency was seen for both males and females. Subjects who reported high levels of pain and discomfort in any dimension (i.e., shoulders, neck, arms, hands, legs, knees, hips, back, or headache) were more frequent in the trauma study population than in the National Health Survey [26].

### Quality of life

Of the 455 who answered all EQ-5D dimension items, 67 (15%) reported no problems (State 11,111) on all five dimensions. Of the 3125 possible EQ-5D health states 174 different states were reported. The median visual analogue scale score on the EuroQol (EQ-VAS) was 70 (IQR: 50–85) and the median EQ index score was 0.745 (IQR: 0.599–0.859). The EQ-5D mean values in different age groups are presented in Table 3, according to recommendations from the EuroQol group. All trauma study values are lower than the Danish population norms [29].

**Table 3:** Mean EQ-5D index score by gender, age group, for current study and Danish population norms

Age group	n	Trauma study value	Danish population norms #	*p*-value
70+				
Male	29	0.741	0.847	0.025
Female	18	0.745	0.818	0.251
60–69				
Male	45	0.588	0.883	0.000*
Female	16	0.718	0.839	0.114
50–59				
Male	61	0.699	0.888	0.000*
Female	24	0.656	0.858	0.000*
40–49				
Male	62	0.691	0.908	0.000*
Female	25	0.636	0.881	0.000*
30–39				
Male	30	0.758	0.928	0.000*
Female	15	0.669	0.903	0.005*
18–29				
Male	82	0.763	0.943	0.000*
Female	48	0.730	0.919	0.000*

n total (completed EQ5D AND PCL): 455

*p < .01 (Two-sample t test with unequal variances)

# ref. no. (38)

The proportion of low EQ index score in this study group was 0.24 (CI: 0.20–0.28) (Table 4). The majority of the variables presented in Table 4 are associated with a high proportion in the low EQ area of the population norm data. There were no difference in proportion of low EQ at the university and the regional hospitals (0.26 vs 0.21, *p* = 0.25) or the two follow-up groups (0.26 vs 0.20, *p* = 0.18). But the severly injured had a higher proportion at the low EQ level (0.21 vs 0.31, *p* = 0.01) for NISS cut at 15. Lower Glasgow Outcome Scale (GOS) scores were associated with a higher proportion of low EQ (Table 4), Mantel-Haenszel chi-square for linear trend = 14.13, (*p* < 0.001).

**Table 4:** QoL and PTSD at follow-up: Characteristics of trauma patients, injury and outcome upon hospital discharge

	Included for analysis n	PTSD Proportions (CI) *n* = 101	Low EQ Proportions (CI) *n* = 108
Total	455	0.22 (0.18–0.26)	0.24 (0.20–0.28)
Hospital trauma level			
University trauma centre	235	0.23 (0.17–0.28)	0.26 (0.20–0.32)
Regional trauma centre	220	0.22 (0.17–0.28)	0.21 (0.16–0.27)
Sex			
Male	309	0.20 (0.16–0.25)	0.24 (0.19–0.29)
Female	146	0.26 (0.19–0.34)	0.24 (0.17–0.32)
Age at trauma - Grouped			
16–39 years	192	0.22 (0.17–0.29)	0.20 (0.14–0.26)
40–59 years	166	0.23 (0.17–0.31)	0.28 (0.21–0.35)
60+ years	97	0.20 (0.12–0.29)	0.25 (0.17–0.35)
Other characteristics			
NISS			
NISS< 15, n	316	0.23 (0.19–0.28)	0.21 (0.16–0.25)
NISS> 15, n	139	0.19 (0.13–0.27)	0.31 (0.24–0.40)
Traffic^a^	258	0.21 (0.16–0.27)	0.22 (0.17–0.28)
ICU^a^	71	0.23 (0.13–0.34)	0.34 (0.23–0.46)
Glasgow Outcome Scale^a^			
GOS 3	37	0.22 (0.10–0.38)	0.38 (0.22–0.55)
GOS 4	97	0.24 (0.16–0.33)	0.29 (0.20–0.39)
GOS 5	280	0.21 (0.16–0.26)	0.18 (0.14–0.23)

CI: 95% confidence interval

*PTSD* by PCL score according to DSM-IV and a PCL summarized score ≥ 37

*QoL* Quality of life

*Low EQ* Trauma patients with EQ value lower than the 2.275 percentile (2 SD below), Danish population specific by age and sex group

^a^n not 455, due to missing data

*ICU* Intensive care unit stay > 2 days

*GOS 3* severe disability, *GOS 4* moderate disability, *GOS 5* good recovery

### Post-traumatic stress disorder

The proportion of patients with PTSD based on the DSM-IV criteria and a PCL summarized score of 37 or higher, was 0.22 (CI: 0.18–0.26). The proportion of patients with PTSD at follow- up was the same in both centres with university centre at 0.23 (0.17–0.28) and (0.22 (0.17–0.28)) at the regional centre. This also applies to the two follow-up groups (0.24 vs 0.19, *p* = 0.216).

The level of trauma showed no differences when compared by centre, gender, or age group (Table 4). It is noteworthy, that the proportion of patients with PTSD was not affected when comparing NISS < 15 0.23 (0.19–0.28) and NISS > 15 0.19 (0.13–0.27) (*p* = 0.345).

## Discussion

Low Qol was seen in 24% of these trauma patients and PTSD in 22% at follow-up. No variation in PTSD was seen for centre (university vs regional) or trauma severity (NISS > 15). This high level of impaired QoL and PTSD points to a significant deficit in complete recovery from serious injury and suggests a potential important public health consequence.

The study population represents the complete clinical population with suspected major trauma,as seen in Scandinavian countries—a major strength of our study. However responders vere older, more often female, had a higher NISS, had a longer ICU stay and had a more severe outcome at end of hospital stay (measured by GOS) than nonresponders. Some of these diffences are well known when using questionnaires, but is still considered a limitation.

Most survivors of major trauma continue to suffer from one or more permanent functional consequences in the long-term. This has a negative impact on their QoL, which often remains far below the general population norms [9, 19, 36]. In both Germany and the United Kingdom, there is consensus about recommending a short-term follow-up and one to two long-term follow-ups after major trauma [37]. However, few trauma registries routinely collect information about long-term follow-up. QoL after severe trauma injury can be drastically changed. Unlike the clinical outcome (mortality) typically measured in trauma trials, QoL reflects the impact of the injury from the perspective of the patient. A better understanding of trauma survivors’ perceptions of their QoL and its influencing factors will assist in developing strategies to improve QoL for trauma patients.

The assessment of PTSD in patients with low NISS vs the PTSD of patients with high NISS showed that the proportions with PTSD were equal. Nevertheless, the two NISS groups differed when looking at the proportion with low QoL. Among patients with severe injury (NISS > 15), the proportion with low QoL was higher than among those with low NISS. Hence, PTSD outcome is not affected by severity measured by NISS, whereas QoL is affected by severity measured by NISS.

PTSD was assessed using the PCL checklist, which is a widely recognized and used self-reporting measure reflecting the DSM-IV definition of PTSD. PCL results may be presented in different ways. As demonstrated, in a Danish setting, the approach depends on the purpose [33]. We combined the diagnostic approach of DSM-IV with a cut-off value, instead of using a cut-off value alone, thereby assuring that all dimensions were represented as stated in the diagnostic criteria. The type of cut-off employed has a major impact on the estimated prevalence of PTSD. The majority of the research in Denmark using the PTSD Checklist Civilian questionnaire has been carried out on military samples [33, 35]. The most frequently used cut-off is 44 for diagnostic purposes, a threshold also used in more extensive Danish studies [34]. Higher cut-off scores have been used in highly traumatized samples, while lower cut-off scores have been suggested for screening use.

A general limitation in injury research is the absence of information on preinjury status. The physical status classification system of the American Society of Anesthesiologists (ASA) was used to classify all trauma patients. No further measurements were used, however, to assess preinjury status. One approach to this issue could simply be to ask the patient. However, the time lag involved in the retrospective collection of data means that the problems in measurement are not thereby reduced. The retrospective nature of our assessment could have led to recall bias, a well-known issue in injury research [38]. Although there is some variation in the population from expected norm values in terms of education, income, etc., we considered that the application of the comparison to population norms was a relevant approach, and more appropriate than intra-individual retrospective assessment. The large differences shown are most likely real differences, and not a consequence of selective injury occurrence with these factors.

The negative effect of injury severity on trauma outcome is obtained in most current international trauma registries [39, 40]. Other effects of injury might be more subtle or might not appear immediately. This study highlights QoL and PTSD as two of the outcomes worth monitoring after the initial treatment of trauma patients, regardless of injury severity score.

## Conclusions

We found that trauma patients from a university and a regional trauma centre showed large proportions of affected QoL and PTSD after 12–24 months in comparison with population norms.

The study supports the understanding that QOL and PTSD are important aspects of patients lives after trauma. Furthermore, the results point to the need for further development and implementation of outcome measures, in terms of both physical and mental health, as well as long-term follow-up on QoL when treating trauma patients. The proportion of patients with evidence of PTSD possibly requiring treatment is 0.22 (0.18–0.26), and the proportion with low QoL compared to the Danish population norm is high 0.24 (CI: 0.20–0.28).

## Abbreviations

ACS: American College of Surgeons
AIS: Abbreviated Injury Scale
ASA: Physical status classification system of the American Society of Anesthesiologists
CI: Confidence interval
DSM-IV: Diagnostic and Statistical Manual of Mental Disorders, 4th Edition
EQ-5D: European Quality of life questionnaire
EQ-5D-5 L: Five-question items, 5 level, in version of EQ-5D
EQ-VAS: European Quality of Life Visual Analog Scale
EuroQoL: European quality of life
IQR: Interquartile range
ISS: Injury Severity Score
NISS: New injury severity score
PCL: PTSD Checklist
PTSD: Post-traumatic stress disorder
QoL: Quality of life
